# The HOME Study: Statistical and economic analysis plan for a randomised controlled trial comparing the addition of Proactive Psychological Medicine to usual care, with usual care alone, on the  time spent in hospital by older acute hospital inpatients

**DOI:** 10.1186/s13063-020-04256-8

**Published:** 2020-05-04

**Authors:** Nicholas Magill, Ian R. White, Jane Walker, Katy Burke, Mark Toynbee, Maike van Niekerk, Fan Yang, Simon Walker, Mark Sculpher, Michael Sharpe, Chris Frost

**Affiliations:** 1 Department of Medical Statistics, London School of Hygiene and Tropical Medicine, London, UK; 2 London Hub for Trials Methodology Research, MRC Clinical Trials Unit at UCL, London, UK; 3 Psychological Medicine Research, Department of Psychiatry, University of Oxford, Warneford Hospital, Oxford, UK; 4 Centre for Health Economics, University of York, Heslington, York, UK

**Keywords:** Randomised controlled trial, Statistical analysis plan, Psychological medicine, Liaison psychiatry, Multi-morbidity

## Abstract

**Background:**

Prolonged acute hospital stays are a problem for older people and for health services. Failure to effectively manage the psychological and social aspects of illness is an important cause of prolonged hospital stay. Proactive Psychological Medicine (PPM) is a new way of providing psychiatry services to medical wards which is proactive, focussed, intensive and integrated with medical care. The primary aim of PPM is to reduce the time older people spend in hospital because of unmanaged psychological and social problems. The HOME Study will test the effectiveness and cost-effectiveness of PPM.

**Methods/design:**

The study is a two-arm, parallel-group, randomised, controlled superiority trial with linked health economic analysis and an embedded process evaluation. The target population is people aged 65 years and older admitted to acute hospitals. Participants will be randomly allocated to either usual care plus PPM or usual care alone. The primary outcome is the number of days spent as an inpatient in a general hospital in the month following randomisation. Secondary outcomes include quality of life, cognitive function, independent functioning, symptoms of anxiety and depression, and experience of hospital stay. The cost-effectiveness of usual care plus PPM compared with usual care alone will be assessed using quality-adjusted life-years as an outcome as well as costs from the NHS perspective.

**Discussion:**

This update to the published trial protocol gives a detailed plan of the statistical and economic analysis of The HOME Study.

**Trial registration:**

ISRCTN registry, ISRCTN86120296. Registered on 3 January 2018.

## Background

In the United Kingdom, National Health Service (NHS) acute hospitals have more than 2 million unplanned admissions of people aged 65 years and older annually. The greater length of stay of older patients means that these admissions account for most (70%) of the available emergency bed days [1]. Excessive time in hospital is bad for patients: it leads to hospital-acquired illnesses, demoralisation and loss of independence after discharge [2]. It is also bad for hospitals because it reduces the availability of beds for other people and increases costs. Strategies to reduce length of stay as well as to reduce admissions are considered to be essential to addressing this problem [3]. A recent review found that, whilst many of the initiatives which aimed to achieve this showed promise, none were of proven effectiveness [4].

The reasons for prolonged hospital stays include not only the complexity of older patients’ medical problems but also inadequately managed psychological and social problems. The psychological problems include psychiatric illnesses such as delirium, dementia, and depression, as well as minor cognitive impairment or anxiety, all of which may slow patients’ discharge from hospital [5, 6]. The social problems include delays in organising post-discharge care arrangements, family members’ expectations or concerns about where the patient will go when leaving hospital, and miscommunications and conflicts about discharge planning within the clinical team. Failure to effectively manage these problems is well documented [7].

We have developed a new way of delivering psychiatry in acute hospitals called Proactive Psychological Medicine (PPM) that aims to address these problems and therefore reduce time spent in hospital. PPM is proactive, takes a broad biopsychosocial approach, provides comprehensive consultant assessment and daily follow-up, and is integrated with the patient’s medical care.

### The HOME Study

The HOME Study is a two-arm, parallel-group, randomised, controlled superiority trial with a linked health economic analysis and an embedded process evaluation. The trial will evaluate the effectiveness and cost-effectiveness of adding PPM to usual care compared with usual care alone. The HOME Study protocol was published previously [8]. This article describes the trial’s statistical and economic analysis plan. This plan has been reviewed and approved by the trial steering committee (TSC) and the data monitoring committee (DMC).

### Research objectives

#### Primary outcome

The main aim of the study is to determine whether adding PPM to usual care affects the time (in days) spent as an acute hospital inpatient in the 30 days post randomisation. Any time spent as an inpatient on a particular calendar date will be counted as a day in hospital.

#### Secondary outcomes

The additional aims of the study are to investigate whether adding PPM to usual care affects the following:

1. Cognitive function, measured by the Montreal Cognitive Assessment–Telephone version (MOCA-T) at 1 and 3 months post randomisation [9]
2. Independent functioning, measured by the Barthel Index of Activities of Daily Living at 1 and 3 months post randomisation [10]
3. Health-related quality of life, measured by the European Quality of Life–5 Dimensions–5 Levels (EQ-5D-5L) questionnaire at 1 and 3 months post randomisation [11]
4. Symptoms of anxiety and depression, each measured by the relevant two items of the Patient Health Questionnaire-4 (PHQ-4) at 1 and 3 months post randomisation [12]
5. Overall quality of life, measured by a trial-specific item (0–10 scale) at 1 and 3 months post randomisation
6. Patient’s experience of hospital stay, measured by a trial-specific item (0–10 scale) at 1 month post randomisation
7. Patient’s view of the length of their hospital stay, measured by a trial-specific item at 1 month post randomisation
8. Discharge destination
9. Secondary healthcare use in the 1 year posts randomisation (including total length of index admission, number of readmissions, and number of days in hospital)
10. Death in the 1 year post randomisation

#### Health economic aims

A further aim is to assess the cost-effectiveness of adding PPM to usual care compared with usual care alone from the NHS perspective. This will be done by measuring the following:

- Quality-adjusted life-years (QALYs), estimated using the EQ-5D-5L measure
- The cost of providing PPM
- The cost of secondary healthcare use

Outcomes will be measured at the time points detailed in Table 1. The table also includes information about how data will be collected.

**Table 1:** Time points and methods of data collection for HOME Study outcomes

Outcome	1 Month (30 days) post randomisation	3 Months (90 days) post randomisation	1 Year post randomisation	Method of data collection
No. of days in hospital in the month (30 days) post randomisation	✓			Routine data/medical records
Cognitive function (MOCA-T)	✓	✓		Patient
Independent functioning (Barthel Index of Activities of Daily Living)	✓	✓		Patient/proxy
Health-related quality of life (EQ-5D-5L)	✓	✓		Patient/proxy
Anxiety and depression symptoms (PHQ-4)	✓	✓		Patient/proxy
Overall quality of life (study-specific item)	✓	✓		Patient/proxy
Experience of hospital stay (study-specific item)	✓			Patient/proxy
View on length of hospital stay (study-specific item)	✓			Patient/proxy
Discharge destination	✓	✓	✓	Routine data/medical records
Secondary healthcare use in the 1 year post randomisation			✓	Routine data/medical records
Death	✓	✓	✓	Routine data/medical records

*Abbreviations: EQ-5D-5L* European Quality of Life-5 Dimensions-5 Levels, *MOCA-T* Montreal Cognitive Assessment–Telephone version, *PHQ-4* Patient Health Questionnaire-4

## Methods

### Trial design

The study is a pragmatic, multicentre, two-arm, parallel-group, randomised, controlled superiority trial with a linked health economic analysis and an embedded process evaluation. The experimental intervention is PPM in addition to usual care delivered by trained clinicians working as members of the medical teams. The comparator is usual care alone. Further details about the trial design can be found in the trial protocol [8].

### Trial treatments

The trial compares two service models: usual care and usual care supplemented with PPM, which has four main components:

1. Early proactive assessment of newly admitted patients using a biopsychosocial approach to identify all the patient’s problems, including psychiatric illness
2. The creation of a systematic management plan to address those problems that pose potential barriers to prompt discharge from hospital
3. Active implementation of this management plan with daily progress reviews
4. Integrated working with ward teams (doctors, nurses, allied health professionals and social care professionals) and out-of-hospital services to ensure that the management plan is implemented

PPM will be delivered at each trial site by a specially trained consultant in psychological medicine/liaison psychiatry and an assisting clinician, working as additional members of the patient’s medical team. Further details can be found in the trial protocol [8].

The comparator treatment is usual care. Usual medical care includes the option for the patient’s medical team to request a consultation from the hospital’s usual liaison psychiatry team.

### Randomisation and blinding

Participants will be allocated to trial arms by stratified randomisation. A database software algorithm for allocating participants was designed by the senior trial statistician. The algorithm allocates participants to usual care plus PPM or to usual care alone in a 1:1 ratio, with stratification done by putative prognostic variables: hospital, sex, and age (65–74, 75–84, ≥85 years). The algorithm is based on the ‘ralloc’ command in Stata software (StataCorp, College Station, TX, USA) and uses random permuted blocks of variable size. The required random seed was selected by the Oxford Clinical Trials Research Unit (OCTRU), which will implement the randomisation system. The participant’s details will be entered into a database via a secure website by the researchers who recruit participants. Their treatment allocation will be automatically generated once the participant’s baseline data have been entered. Neither study researchers nor participants will therefore be able to predict treatment allocation. A study researcher will inform the patients of their allocation and will inform PPM teams about participants who have been allocated to usual care plus PPM.

The staff who collect outcome data and the study statisticians will be blinded to participants’ allocated interventions. Study statisticians will carry out analyses with trial arms described only as ‘A’ and ‘B’. HOME Study researchers who recruit participants will carry out the randomisation procedure described above and therefore will not be blinded to allocation, nor will it be possible to blind participants and their clinicians, owing to the nature of the study interventions.

Full details of randomisation and blinding are stored on a University of Oxford–based server with confidential access restricted to the OCTRU statistics team. Trial statisticians do not have access to this, because they are blind to treatment allocation. Monitoring of the randomisation system is also being undertaken by the OCTRU statistics team.

### Sample size

A total of 3588 participants are required to detect a reduction of 1 day (from 9 to 8 days; standard deviation, 9) in the mean number of days spent in hospital with 90% power at the 5% significance level, using a two-tailed weighted *t* test with weights as specified to be used in the primary analysis and allowing for 5% loss to follow-up. The value of the expected standard deviation was obtained from pilot data.

A series of measures will be taken aimed at achieving the target sample size:

- Researchers will be embedded in clinical teams at each study centre.
- Screening of patients will be used to obtain a representative sample of the relevant population and to give all potentially eligible patients the opportunity to participate.
- Researchers will be trained in how to explain the study to patients who are unwell and to their carers.
- Multiple wards will be used for recruitment at each study centre.
- The trial management group (TMG) will monitor recruitment weekly to ensure identification of problems and implement solutions.

### Outcome data collection

Data describing the participant’s hospital stay, discharge destination, subsequent hospital admissions, secondary healthcare use and mortality will be obtained from national datasets of routinely collected clinical data and from local hospital records and datasets. At 1 month (30 days) and 3 months (90 days) post randomisation, a member of the research team will contact the participant (or an appropriate proxy) to administer the study questionnaires, either by telephone or face to face. All members of the research team will be trained in the standard operating procedures for the tasks they are allocated, and their competency will be assessed by the central trial team. The majority of the secondary outcome data will be collected by telephone by the central trial outcome team.

The measures that will be taken to minimise missing data include the following:

- For the primary outcome, we will use routinely collected NHS clinical data.
- For the secondary outcomes we will:
  -  ◦ Obtain full contact details from participants as well as a back-up ‘best contact’ address (i.e., contact details of a friend/relative nominated by the participant)
  -  ◦ Record participants’ discharge destination from hospital
  -  ◦ Collect data from proxies when participants are unable to give reliable data
  -  ◦ Use reminder telephone calls and letters
  -  ◦ Check with the patient’s general practitioner regarding whether they are alive and/or have moved their address before collecting outcomes

### Statistical interim analysis, data review and stopping guidelines

The HOME Study has a DMC, which will monitor trial data and make recommendations to the TSC on whether there are any ethical or safety reasons why the study should not continue. DMC members will act independently of the TSC, TMG and funder. The DMC will monitor data at two interim assessment time points (i.e., analysis will be done at three time points in total, including the final assessment) and will receive a report from the trial statisticians, who will attend only by invitation. The assessment times are defined as the time point at which 1200 (approximately one-third), 2400 (approximately two-thirds) and 3588 patients (the final sample size) have been recruited to the trial and have been followed up for30 days.

In open sessions, the DMC will review summaries of participants’ baseline characteristics (both trial arms combined), including the following:

- Hospital where the participant was recruited (randomisation stratifier)
- Sex (randomisation stratifier)
- Age (randomisation stratifier)
- Number of participants in the strata (i.e., the 24-category combination of age [3 groups], sex [2 groups] and hospital [up to 4 groups])
- Cognitive function (MOCA-T; a secondary outcome)
- Independent functioning (Barthel Index of Activities of Daily Living; a secondary outcome)
- Health-related quality of life (EQ-5D-5L; a secondary outcome)
- Depression and anxiety symptoms (PHQ-4; a secondary outcome)
- Overall quality of life (study-specific item; a secondary outcome)

The DMC will also monitor the data completeness (proportion of complete data) of secondary outcome data at the 1- and 3-monthfollow-up time points. Participants will be placed into one of three categories: (1) complete data obtained; (2) partial data obtained; or (3) no data obtained, with reasons listed for those in this final category. Data completeness will not be assessed by trial arm in open sessions.

In closed sessions, the DMC will monitor semi-blinded data, which is to say that the trial arms will be labelled as ‘A’ and ‘B’. In these sessions, the DMC will review the number of participants randomised to each stratum, baseline characteristics, and data completeness, by trial arm. The DMC will also monitor the occurrence of serious adverse events (SAEs) and (if unblinded) suspected unexpected serious adverse reactions (i.e., SAEs that are likely to be due to the implementation of PPM). The DMC will focus on the number of participant deaths that occur within 30 days of study enrolment. In these sessions, the number of participants randomised to each stratum, baseline data, and data completeness will be monitored by trial arm.

Interim analyses of the primary outcome data will not be undertaken, because these require data that will not be available during the relatively short recruitment period. There are therefore no statistical stopping rules for benefit in this study; the DMC will recommend stopping only on safety grounds. The trial statistician will provide tabular summaries of all-cause mortality by semi-blinded trial arm (A/B) and by the randomisation stratifiers. The DMC statistician member will use Fisher’s exact test to assess the null hypothesis that the all-cause mortality rates in the two trial arms are the same. To address multiplicity concerns about repeatedly testing at every interim assessment time point, the O’Brien-Fleming sequential stopping method will be implemented. This requires application of Fisher’s exact test with *P* values for statistical significance set at 0.0005, 0.014 and 0.045 at the first, second and final assessment time points, respectively [13]. The test at the final assessment will not be used to stop the trial; it will be used only to assess the statistical significance of the difference seen at the final analysis. Tests will be two-sided. If a statistically significant difference between trial arms in all-cause mortality is detected, a clinical subgroup of the DMC will be unblinded to allocation status and will determine, through case note review, whether PPM could have led to any of the excess deaths.

### Timing of final analysis

The final analysis will be completed once all data have been collected and the database has been locked. All outcomes and time points will be assessed at the same time.

### Blinded analysis

All statistical analyses will be carried out with trial arms described only as ‘A’ and ‘B’.

### Statistical properties

#### Statistical significance and multiple testing

The study aims to determine whether adding PPM to usual care reduces the time (days) spent by patients in acute hospitals in the 1 month post randomisation. Consequently, there is only one primary outcome and no multiple testing in the main effectiveness analysis. Hypothesis tests relating to the secondary outcomes are considered to be exploratory. Therefore, the significance level used will be 0.05, and 95% confidence intervals will be reported. The one exception to this will be the analysis of treatment effect on deaths (secondary outcome 10). This outcome will be the subject of interim analyses conducted by the DMC, for which the O’Brien-Fleming sequential stopping rule is being used [13]. The planned test level at the final assessment time point is 0.045. The DMC will make multiple tests of the null hypothesis that there is no difference in deaths between the trial arms. Adjustment has been made for the inflation of the type I error which is a consequence of these multiple tests.

#### Definition of analysis populations

The trial is designed primarily to assess effectiveness (i.e., the effect of treatment in everyday conditions) [14]. It will also assess efficacy for the primary outcome. All analyses of effectiveness will follow the intention-to-treat (ITT) principle, which states, ‘The effect of a treatment policy can be best assessed by evaluating on the basis of the intention to treat a subject (i.e. the planned treatment regimen) rather than the actual treatment given’ [15]. Effectiveness analysis populations will be defined as the ITT population, and all randomised participants will be included in their randomised groups. For these analyses, participants will be analysed according to the group to which they were randomised and not according to the intervention they actually received.

An efficacy analysis of the primary outcome will be carried out using per-protocol analysis and will use covariate adjustment (covariates of age, centre, and sex) with the aim of minimising selection bias. Using the definition of treatment receipt that will be described later, this analysis will exclude those participants who are recorded as not having received treatment; that is, outcomes will be compared between randomised treatment groups for a subset of the ITT population. This subset will include those PPM arm participants who receive the intervention and all usual care arm participants. This analysis will unblind the trial statisticians and will therefore be carried out once all other analyses have been completed.

### Trial population and descriptive analyses

#### Eligibility

Participants will be recruited from the acute wards (not emergency departments) of Oxford University Hospitals NHS Foundation Trust, Royal Devon and Exeter NHS Foundation Trust, and Cambridge University Hospitals NHS Foundation Trust. To be included in the trial, patients must

- Be aged 65 years or older
- Be an inpatient in an acute ward where trial recruitment is taking place
- Have been admitted non-electively (i.e., their hospital admission was unplanned)
- Be expected by their clinical team to remain an inpatient for at least 2 days from the time of trial enrolment
- Be able to give informed consent or, if unable to give consent, a consultee advises that trial participation is appropriate

Patients will be excluded if at the time of enrolment

- They are moribund, which is defined for this trial as the clinicians caring for the patient estimating that they are likely to die before discharge from hospital
- Their participation in the trial is judged to be clinically or practically inappropriate (e.g., the patient is not from the local area served by the hospital)
- They have already been enrolled in the trial
- They have already been referred to the usual care liaison psychiatry team
- They have already been a general hospital inpatient continuously for 1 week
- They do not read or speak English

#### Representativeness of study sample and patient throughput

**Fig. 1:**
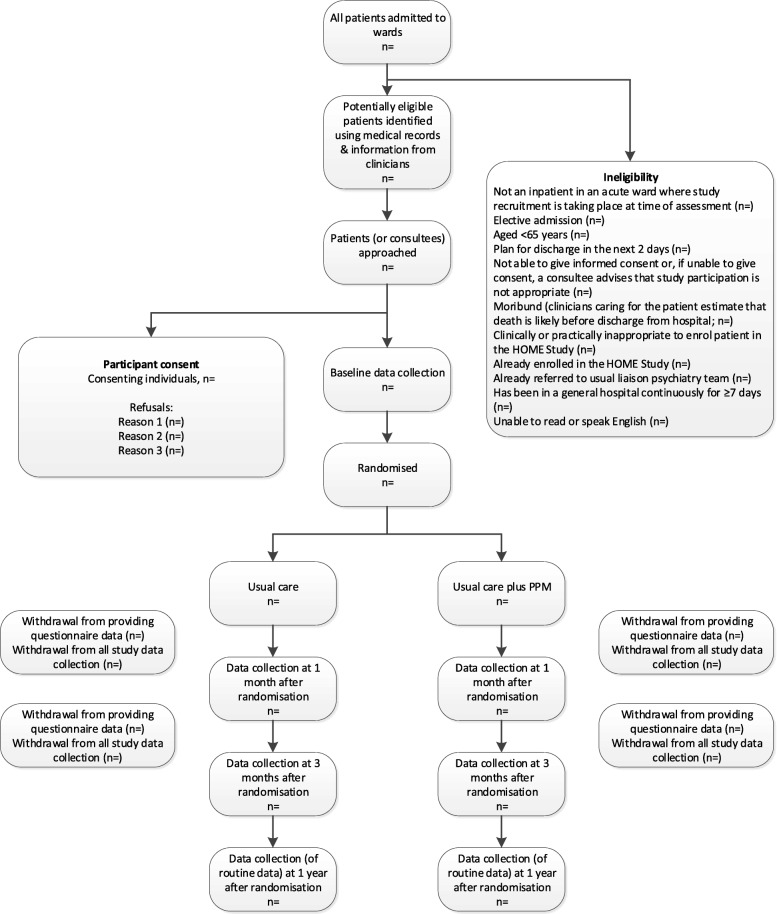
Consolidated Standards of Reporting Trials (CONSORT) diagram of the flow of participants through the trial

The flow of participants through the trial will be summarised as a Consolidated Standards of Reporting Trials (CONSORT) diagram, as shown in Fig. 1.

#### Withdrawal from treatment and/or follow-up

It is anticipated that some participants may withdraw from data collection. This may involve withdrawal from providing questionnaire data or withdrawal of consent for the collection of any data (both questionnaire data and data collection from healthcare records and relevant databases). Patients (or their representatives on their behalf) may decide, during their participation in the study, to refuse relevant interventions; these refusals will be noted but will not be considered withdrawals from the study. Withdrawals and loss to follow-up, together with reasons, will be reported by trial arm. Any deaths (and their causes) will be reported separately.

#### Baseline comparability of randomised groups

The following data will be collected at baseline:

- Hospital where the participant is recruited
- Age
- Sex
- Ethnicity (white British; white Irish; any other white ethnic background; white and black Caribbean; white and black African; white and Asian; any other mixed background; Indian; Pakistani; Bangladeshi; any other Asian background; Caribbean; African; any other black background; Chinese; any other ethnic group; not stated)
- Relationship status (spouse/partner; no spouse/partner)
- Usual place of residence (private residence; care home or nursing home; other)
- Whether admitted from usual place of residence (yes; no)
- If not admitted from usual residence, where the participant was admitted from (other hospital; private residence, not patient’s own; care home/nursing home, not patient’s normal home; other)
- Index of Multiple Deprivation based on home postcode
- Rural/urban classification of the participant’s home based on postcode (urban major conurbation; urban minor conurbation; urban city and town; urban city and town in a sparse setting; rural town and fringe; rural village; rural hamlets and isolated dwellings; rural town and fringe in a sparse setting; rural village in a sparse setting; rural hamlets and isolated dwellings in a sparse setting)
- Whether the participant lives alone (yes; no)
- Employment status (working; retired; not working due to health; not working for another reason)
- Reason for hospital admission (recorded as free text and to be summarised using a categorisation that will be formed)
- Diagnoses (medical and psychiatric) recorded on admission
- Medication prescribed
- Days in hospital prior to enrolment, meaning days spent in hospital between admission and randomisation, not counting the day of randomisation itself (e.g., if someone was admitted at some time the day before being randomised, this would count as 1 day)
- Cognitive function (MOCA-T)
- Independent functioning (Barthel Index of Activities of Daily Living)
- Health-related quality of life (EQ-5D-5L)
- Depression and anxiety (PHQ-4)
- Overall quality of life (study-specific item)
- Secondary healthcare use (including number of admissions to hospital) in the year prior to randomisation

These baseline characteristics will be reported by trial arm and for both arms combined. Numbers (with percentages) for categorical variables and means (and standard deviations) or medians (with lower and upper quartiles) for continuous variables will be presented. There will be no tests of statistical significance or confidence intervals for differences between randomised groups on any baseline variable.

#### Description of receipt of intervention

Receipt of intervention is defined as having occurred when a PPM clinician has reviewed the participant’s case and completed an assessment and an action plan for them. The completion of the minimum requirement will be summarised within the PPM trial arm using absolute and relative frequencies. Other elements of the interventions given will also be described. The PPM consultation will not be available to any participant in the usual care trial arm, so no participant in that arm will be excluded from the per-protocol analysis.

### Analysis

#### Outcome definitions

##### Primary outcome

Time (days) spent by participants in acute hospitals in the 30 days post randomisation. A day in hospital is defined as a patient spending any time in hospital on a particular date. If this participant remains in hospital for even a short period of time after midnight on the next calendar date, this is defined as an additional day spent in hospital.

##### Secondary outcomes

1. Cognitive function measured using the MOCA-T at 1 and 3 months post randomisation [9]; we will use standard scoring [16].
2. Independent functioning measured using the Barthel Index of Activities of Daily Living at 1 and 3 months post randomisation [10]; we will use standard scoring [17].
3. Health-related quality of life measured using the EQ-5D-5L at 1 and 3 months post randomisation [11]. Responses to the five items of the EQ-5D-5L will be transformed into a health-related quality of life score using two methods: (1) van Hout et al.’s crosswalk algorithm [18] and UK EQ-5D-3L value set [19] and (2) the UK EQ-5D-5L value set [20]. The former of these represents the National Institute for Health and Care Excellence’s preferred method [21].
4. Symptoms of anxiety and depression measured by the PHQ-4 at 1 and 3 months post randomisation; we will use standard scoring [12].
5. Overall quality of life measured using a study-specific item at 1 and 3 months post randomisation. This item is measured on an interval scale where scores range between 0 and 10. Higher scores indicate better quality of life.
6. Participants’ experience of hospital stay measured using a study-specific item at 1 month post randomisation. This item is worded: ‘Thinking about your recent hospital stay, on a scale of 0–10, where 0 is terrible and 10 is excellent, how would you rate the care you received in hospital’?
7. Participants’ views on the length of their hospital stay as measured using a study-specific item at 1 month post randomisation. The item is worded thus: ‘Thinking about your recent hospital stay, what do you think about your stay in hospital – was it too short, about right, or too long’?
8. Discharge destination. This outcome is measured at 1 month, 3 months and 1 year post randomisation. Discharge destination is coded as follows: A (private residence, patient’s own); B (private residence, not patient’s own); C (care home/nursing home, patient’s normal home); D (care home/nursing home, temporary placement); E (care home/nursing home, acute hospital bed); F (community hospital); G (hospice); H (psychiatric hospital); or I (other). The outcome will be coded as a dichotomous variable whose levels are private residence (levels A and B above) and not private residence (levels C, D, E, F, G, H and I above).
9. Secondary healthcare use in the 1 year post randomisation, including total length (days) of index admission post randomisation, number of readmissions, and number of days in hospital.
10. Deaths in the 1 year post randomisation.

### Statistical analysis methods

All outcomes will be described in tabular format by trial arm and time point. Discharge destination will be summarised by its nine levels rather than by the dichotomisation used for inferential analysis. Means and standard deviations as well as medians and interquartile ranges will be reported for all continuous outcomes (including time-to-event outcomes). Categorical outcomes will be described using absolute and relative frequencies.

The treatment effect for the primary outcome (number of days spent in hospital in the 30 days post randomisation) will be estimated using a linear regression model. The model will include (1) centre (Cambridge, Exeter or Oxford) by treatment interaction terms, (2) stratification factors (hospital, sex and age, which will be treated as continuous in the analysis model) as fixed effects, and (3) wards as fixed or random effects (the final choice being dependent on the number of wards included). The effect of treatment on the primary outcome will be a weighted mean of the three centre-specific treatment effects, with weights proportional to the number of people randomised at each centre. As a check on the robustness of results to normality assumptions, non-parametric bootstrap methodology (bias corrected and accelerated, 2000 replications, with allowance for stratification) will be used to construct the confidence interval. The difference between the means together with a 95% confidence interval will be reported.

Secondary outcome numbers 1, 2, 3, 4 and 5 (MOCA-T, Barthel, EQ-5D, anxiety and depression subscales of PHQ-4, study-specific measure of overall quality of life) are recorded at baseline, 1 month post randomisation and 3 months post randomisation. Treatment effects will be estimated using analysis of covariance; that is, post-randomisation measures will be included in the outcome vector, and baseline measures will be treated as covariates. Data will be arranged in wide format (one participant per row), and treatment effects at 1 and 3 months post randomisation will be estimated using separate models. This is to allow the use of multiple imputation (MI) (see the next section on missing data). Models will include the fixed effects that were described in the model for the primary outcome together with a fixed effect for outcome measured at baseline. The estimated effect of treatment will be a weighted mean of the three centre-specific treatment effects at each of the post-randomisation time points. As a check on the robustness of results to normality assumptions, confidence intervals will be constructed from bootstrap samples that will be drawn from each of the multiply imputed datasets (2000 replications, with allowance for stratification) [22]. The limits of the 95% confidence interval will be calculated using the 2.5 and 97.5 percentiles of treatment effect estimates across all bootstrap samples and across all multiply imputed datasets.

Experience of hospital stay (secondary outcome number 6) is continuous and measured at 1 month post randomisation. Note that this outcome is not measured at baseline. Models for this outcome will use linear regression and will include the same fixed effects as the model for the primary outcome. This outcome is patient-reported with an expectation that there will be a considerable amount of missing data (partly due to participants dying). MI will be used to address this (see the next section on missing data). As a check on the robustness of results to normality assumptions, confidence intervals will be constructed from bootstrap samples that will be drawn from each of the multiply imputed datasets (2000 replications, with allowance for stratification) [22].

Secondary outcome number 7 (patient’s view on length of hospital stay) will be modelled using ordered logistic regression, provided that there is no evidence that the proportional odds assumption does not hold. If the assumption appears broken, the outcome will be modelled using multinomial logistic regression. This outcome is patient-reported with an expectation that there will be a considerable amount of missing data (partly due to participants dying). MI will be used to address this (see the next section on missing data). Secondary outcome number 8 (discharge destination) will be modelled using logistic regression. Covariates will be the same as those included in the model for the primary outcome. Analysis will be conditional on the participant being admitted from a private residence and not being dead when leaving; that is, the model will be fitted to a subset of the sample based on this information. The effect of treatment offer on the outcome will be calculated in the same manner as for the primary outcome (i.e., as a weighted mean of the three centre-specific treatment effects).

Secondary outcome number 9 (secondary healthcare use in the 1 year post randomisation) will be modelled using a number of approaches. Total length (days) of index admission post randomisation will be handled as a time-to-event outcome. The Cox proportional hazards model, with censoring for deaths, will be fitted in order to estimate the effect of treatment. Number of readmissions will be treated as count data, and therefore the treatment effect will be estimated using a Poisson model with robust standard errors to allow for likely overdispersion and non-independent events. Total time (days) in hospital will be handled as a continuous outcome, and the effect of treatment will be estimated using a model similar to that used for the primary outcome. Confidence intervals for this model will be estimated by bootstrapping due to the fact that these outcomes are expected to be skewed and truncated. For all three of these models, covariates will be the same as those included in the model for the primary outcome. The effect of treatment offer on the outcome will be calculated in the same manner as for the primary outcome (i.e., as a weighted mean of the three centre-specific treatment effects).

Secondary outcome number 10 (deaths) will be modelled using survival analysis. Kaplan-Meier curves will be used to plot survival over time by trial arm. The Cox proportional hazards model will be used to estimate the effect of treatment on outcome. Covariates will be the same as those included in the model for the primary outcome. The effect of treatment offer on the outcome will be calculated in the same manner as for the primary outcome (i.e., as a weighted mean of the three centre-specific treatment effects).

### Missing data including deaths

In the main analysis of the primary outcome, a patient who dies on a particular day has the same outcome as a patient who leaves hospital on that day; hence, this can be interpreted as a hospital-centred analysis. Two supplementary analyses of this outcome will be used to estimate the treatment effect, and these will take more participant-centred interpretations of death.

For the first supplementary analysis, a participant’s outcome will be constructed as the time (days) he/she spent in hospital in the 30 days post randomisation as a proportion of the time (days) that the participant was alive during those 30 days. For example, if a participant died at the end of spending 10 continuous days in hospital, the outcome would count as 1. If another participant spent 10 days in hospital and then died after a further 10 days, his/her outcome would be 0.5. The model for this analysis will include the same fixed effects described in the main model for the primary outcome. Outcomes will be weighted by how long participants were alive in the 30 days post randomisation. As a check on the robustness of results to normality assumptions, non-parametric bootstrap methodology (bias corrected and accelerated, 2000 replications, with allowance for stratification) will be used to construct the confidence interval.

For the second supplementary analysis, the treatment effect will be estimated using time (days) spent by participants in acute hospitals in the 30 days post randomisation amongst only those participants who survived to 30 days post randomisation. The model for this supplementary analysis will include the same fixed effects described in the main model for the primary outcome. The analysis model will be conditional on survival until 30 days post randomisation; that is, the model will be fitted to a subset of participants alive at this time. Treatment effects will be weighted means of the three centre-specific treatment effects with weights proportional to the number of people randomised at each centre. As a check on the robustness of results to normality assumptions, non-parametric bootstrap methodology (bias corrected and accelerated, 2000 replications, with allowance for stratification) will be used to construct the confidence interval.

For patient-reported outcomes, an appreciable amount of missing data is expected. This may be for a number of reasons, such as participants being out of contact, being too ill to complete questionnaires, or due to death. Two levels of data missingness are anticipated: missing questionnaire items and missing outcome values. Missing scale items will be addressed using individual mean imputation, provided that 20% or less of items are missing for a given participant. Specifically, this involves calculating the within-participant mean of the non-missing values for a particular questionnaire. This mean is then used to impute the missing value(s), provided that the number of missing items for that participant is small. It has been shown that at low proportions of missing items, the correlation between imputed and true values is high, and there is no additional benefit of using more sophisticated methods such as MI [23]. In addition, missing outcome values are anticipated, some of which will be due to the deaths of participants. It is considered to be conceptually problematic to regard the outcomes of those who die before the end of follow-up as properly missing in a data modelling process. This is because it is difficult to make any assumption or draw any inference about a person’s health state if they are not alive. Multiple imputation by chained equations (MICE) provides a method for valid estimation of treatment effects without making assumptions about levels of outcomes for those who have died. The method involves three steps. In the first step, missing data are replaced by simulated values drawn from the predicted distribution of missing data conditional on observed data. This involves fitting a model to the observed data, simulating a random draw of the model parameters from their posterior distribution, and simulating random draws of the missing data from this model. This is done a number of times, thereby generating a number of imputed datasets. In the second step, models are fitted to each imputed dataset (providing estimated treatment effects). In the third step, parameter estimates from multiply imputed datasets are combined. Any variables and interaction terms used in the analysis model (second step), together with any predictors of missingness, must be included in the imputation (first) step. Imputation models will include any variables in the main model (as listed in the previous section), values of the variable being analysed at other time points, and auxiliary variables. Auxiliary variables are considered to be anything that is correlated with the incomplete variable and is sometimes observed when the incomplete variable is missing. These variables will include the following baseline demographic and clinical variables that are considered to be possibly correlated with incomplete outcomes:

- Usual place of residence
- Whether the participant was admitted from their usual place of residence
- Where the patient was admitted from
- Relationship status
- Employment status
- Whether the patient lives alone
- Deprivation score
- Rural/urban classification

The list of auxiliary variables will also include the primary outcome and secondary outcomes derived from routine data/medical records (measured at baseline and both post-randomisation time points). This implies that baseline measurements of outcomes for those who die will be allowed to contribute to the imputation process. However, as mentioned above, the analysis will make no assumptions about the levels of outcomes following death. For this reason, imputed values for those participants who die before the end of follow-up will be discarded before the analysis step. For each of secondary outcomes 1–7, 100 multiply imputed datasets will be generated. Imputation will be done separately for the two randomised groups. For EQ-5D-5L, MICE will be used to impute EQ-5D-5L values for alive participants, and if the missingness is due to death, the EQ-5D-5L values are set to be 0.

The main analysis of those outcomes collected from routine data/medical records will use all available data and assume that missing data are missing at random. In the event that there is substantial missing data (> 10% missing observations) or substantial imbalance in missing data between arms (difference in proportion of missing observations between trial arms > 10 percentage points) for these outcomes, the use of MI with auxiliary variables included in the imputation step will be considered. For the outcomes collected from routine data/medical records, missing data due to death will be handled in a number of ways, some of which have already been described. For example, total length of index admission (part of secondary outcome number 9) will be treated as a time-to-event outcome with censoring for death. In addition, discharge destination will be modelled only for those participants who survived until discharge from hospital. Total number of readmissions in the year following randomisation (part of secondary outcome number 9) will be modelled in two ways: using a count of readmissions and using the number of readmissions scaled by time alive (measured in years). Total time (days) spent in hospital in the 1 year following randomisation (part of secondary outcome number 9) will also be modelled in two ways: using the total number of days participants spent in hospital and using the total number of days participants spent in hospital as a proportion of time alive.

### Pre-specified subgroup analysis

Pre-planned subgroup analyses are planned using the randomisation stratification variables (hospital, sex, and age groups) because it is anticipated that these are the most likely effect modifiers. The models will be fitted for the primary outcome using the same population as the other effectiveness analyses (i.e., ITT population). The models will include interactions terms between trial arm and each of the stratification variables.

### Supplementary/additional analyses and outcomes

A per-protocol analysis will be carried out for the primary outcome, as described earlier. This will be done by fitting a model similar to that used for the primary outcome. The difference will be that instead of using trial arm as the exposure variable of interest, this variable will be treatment receipt. The construction of this variable was described earlier.

In addition, supplementary analyses will examine the effect of data being collected from either patients or proxies. Some secondary outcome data (Barthel Index of Activities of Daily Living, EQ-5D-5L, PHQ-4, overall quality of life, experience of hospital stay, view on length of hospital stay) will be collected from proxies when participants are unable to provide data. The following analyses will be performed:

- We will examine whether the occurrence of proxy measurements differs materially by treatment arm.
- As a sensitivity analysis, we will fit an interaction between proxy/non-proxy and treatment and allow different variances for proxy/non-proxy.

The primary analysis will ignore whether data were collected from patients or proxies, because the person from whom data is collected may be influenced by treatment.

### Health economics and cost-effectiveness

The health economics analysis aims to assess the cost-effectiveness of usual care plus PPM with usual care alone. The analysis will take the perspective of the NHS. Costs will be expressed in UK pound sterling (GBP) at 2019/2020 prices, and health outcomes will be expressed in QALYs, in line with current UK guidance for economic evaluations [24]. For the base case, cost-effectiveness will be assessed over the 1-year trial period. If there are found to be differences over this period which may result in the cost-effectiveness results being expected to differ over the longer term, extrapolation of trial results will be conducted. Cost-effectiveness results will be expressed in terms of incremental cost-effectiveness ratios (ICERs) and incremental net health benefits (NHBs) at thresholds of £13,000 [25], £20,000 and £30,000 per QALY [26]. If extrapolation is conducted, costs and QALYs beyond 1 year will be discounted at 3.5% per annum in line with UK recommendations [26].

### Resource use and costs

Resource use and costs in secondary care and intervention costs will be estimated as part of the analysis. Secondary healthcare use in the 1 year post randomisation will be recorded using routine data (hospital episode statistics; secondary outcome number 9). Liaison psychiatry resource use (in both trial arms) will be estimated using information from participants’ medical records. Staff resources associated with PPM (in intervention arm only) will be estimated on the basis of receipt of intervention. Using NHS reference unit costs [27], Personal Social Services Research Unit Cost of Health and Social Care [28], costs of healthcare resource use, costs of intervention, and total costs will be calculated for all patients in the trial.

### Health-related quality of life

QALYs will be the health outcome used in the cost-effectiveness analysis. Health-related quality of life measured by the EQ-5D-5L (secondary outcome number 3) is recorded at baseline and at 1 month and 3 months post randomisation. Responses to EQ-5D-5L are transformed into health-related quality of life weights using two different methods (using van Hout et al.’s crosswalk algorithm [18] and UK EQ-5D-3L value set [19] or the new UK EQ-5D-5L value set [20]). Consistent with the main analysis, the base case analysis will pool the EQ-5D-5L assessed by patients themselves and proxies together. Other approaches to handling the patient- and proxy-ratedEQ-5D-5L will be explored in a sensitivity analysis. Patients will be assumed to experience constant health-related quality of life from 3 months onwards until death or 1 year. Death within the 1-yearfollow-up duration is informed by UK Office for National Statistics mortality data (secondary outcome number 10). The health-related quality of life weights and survival data will be combined to estimate QALYs over the 1-year period, based on the area under the curve method and linear interpolation between time points [29], for all patients.

### Missing data

Where costs and EQ-5D scores are missing, MI will be performed to replace each missing observation with a set of imputed values following the method recommended by Faria et al. for the imputation of economic data [22]. Predictive mean matching will be used to ensure that imputed values are in the appropriate range (e.g., no negative costs or EQ-5D scores greater than 1). We will use MICE, and Rubin’s rules [30] will be implemented for the subsequent analysis of multiple datasets. All analyses will be conducted using Stata software.

### Analysis

In the within-trial analysis, costs and QALYs will be calculated per patient and then analysed using both generalised linear models and seemingly unrelated regression models controlling for covariates to estimate the incremental mean costs and QALYs of adding PPM to usual care [31]. Covariates considered will be the same as for the main statistical analysis, including the centre (Cambridge, Exeter, or Oxford) by treatment interaction terms and baseline health-related quality of life weight for QALYs [32]. ICERs and incremental NHBs will be calculated at thresholds of £13,000, £20,000 and £30,000 per QALY. The probability of usual care plus PPM compared with usual care being cost-effective at different cost-effectiveness thresholds will be calculated [33, 34] and represented visually as a cost-effectiveness acceptability curve [35, 36]. Analyses will also be performed for the pre-defined subgroups. A subgroup analysis will be conducted for three centres to explore the centre-specificcost-effectiveness.

If differences in costs or outcomes between usual care plus PPM compared with usual care alone are found over the trial period, which would be expected to differ over the longer term, extrapolation of the trial results will be conducted. A decision analytic model will be developed to capture the costs and QALYs over an appropriate time horizon (the time over which costs and QALYs could be expected to differ between the management strategies, which may be lifetime) [29, 37]. The model structure will be developed with clinical input and will synthesise data from the trial with other external sources to estimate cost-effectiveness. Uncertainty in the parameters in the model will be reflected using probability distributions, with the resulting overall decision uncertainty presented using cost-effectiveness acceptability curves [34].

### Specification of statistical packages

All analyses will be performed using Stata software.

## Data Availability

Not applicable.

## Abbreviations

DMC: Data monitoring committee
EQ-5D-5L: European Quality of Life-5Dimensions-5 Levels
HRQoL: Health-related quality of life
ICER: Incremental cost-effectiveness ratio
ITT: Intention to treat
MI: Multiple imputation
MICE: Multiple imputation by chained equations
MOCA-T: Montreal Cognitive Assessment–Telephone version
NHB: Net health benefit
NHS: National Health Service
OCTRU: Oxford Clinical Trials Research Unit
PHQ-4: Patient Health Questionnaire-4
PPM: Proactive Psychological Medicine
QALY: Quality-adjusted life-year
SAE: Serious adverse event
TMG: Trial management group
TSC: Trial steering committee
